# Design of melt-recyclable poly(ε-caprolactone)-based supramolecular shape-memory nanocomposites†

**DOI:** 10.1039/c8ra03832e

**Published:** 2018-07-30

**Authors:** Florence Pilate, Zhi-Bin Wen, Farid Khelifa, Yan Hui, Sebastien Delpierre, Luo Dan, Rosica Mincheva, Philippe Dubois, Ke-Ke Yang, Jean-Marie Raquez

**Affiliations:** Laboratory of Polymeric and Composite Materials (LPCM), Center of Innovation and Research in Materials and Polymers (CIRMAP), University of Mons (UMONS) 23 Place du Parc 7000 Mons Belgium jean-marie.raquez@umons.ac.be; Center for Degradable and Flame-Retardant Polymeric Materials (ERCEPM-MoE), National Engineering Laboratory of Eco-Friendly Polymeric Materials (Sichuan), State Key Laboratory of Polymer Materials Engineering, Sichuan University Chengdu Sichuan 610064 China

## Abstract

A novel poly(epsilon-caprolactone) (PCL) supramolecular network exhibiting shape-memory behavior was successfully constructed with pendant UPy units that are highly able to dimerize. The dynamic network was obtained by a simple and versatile strategy consisting of chain-extension reaction between α,ω-dihydroxyoligoPCL and hydroxylated UPy units in the presence of hexamethylene diisocyanate as a coupling agent and further intermolecular dimerization of the UPy along the polyurethane backbone. ^1^H NMR analyses confirmed the dynamic features of the system, and DMTA in tensile mode was investigated to assess the SMP properties. Recyclability was also assessed by taking advantage of these supramolecular networks. Further addition of cellulose nanocrystals into the polymer network enabled adjustment of the extent of the net-points and therefore the SMP features. As confirmed by dispersion tests in solution and SEM observations, these bio-based nanofillers were homogeneously distributed in the network *via* supramolecular interaction between the hydroxyl groups present on their surface and UPy moieties along the polyurethane backbone. Thus, the here developed nanomaterials might reveal applicability in areas where a combination of SMP and biocompatibility is needed.

## Abstract 2

Novel melt-recyclable poly(ε-caprolactone)/cellulose nanocrystals supramolecular nanocomposite networks with shape-memory behavior have been successfully constructed by playing with UPy chemistry.

## Graphical abstract

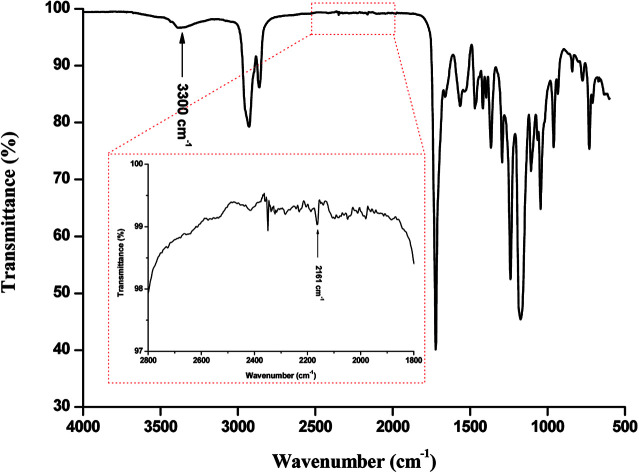

## (1) Introduction

Shape-Memory Polymers (SMPs) have been gaining great interest as a new class of stimuli-responsive materials for 30 years due to their high elastic deformation, low density, relatively low cost, easy processing, high recoverable strain within a large range of stimuli and chemical stability as well as potential applications ranging from biomedical materials to engineering thermoplastics.^1^ These polymeric materials can be fixed into a metastable shape and, subjected to an external stimulus such as variation in temperature, light, magnetic field and water or solvent immersion, will recover their original shape.^2,3^ The most common SMPs, *i.e.*, dual-shape heating-responsive SMPs, are built on a network architecture composed of permanent and switching domains: they combine chemical or physical covalent crosslinks defining the permanent domain, and switching domains associated with a transition temperature *T*_trans_ (glass temperature (*T*_g_) or melting temperature (*T*_m_)). In this case, the deformed temporary shapes are stabilized by a cooling step below the *T*_trans_ and the polymer chains are quenched in a stressed state. A subsequent heating above the *T*_trans_ allows for entropic relaxation of the freezed macromolecular chains, leading to permanent shape recovery. Interestingly, some semi-crystalline polymers also display an exciting reversible elongation-induced crystallization in the presence or in the absence of an external stress,^4^ resulting in SMPs with reversible properties, *i.e.*, the two-way SMPs. However, in the case of irreversibly cross-linked semi-crystalline materials, the crystalline features of the resulting network (*T*_m_, crystallinity degree and crystallization rate) are depressed compared to the precursor linear material due to the reduction of the molecular motion and crystal growth.^5–7^

Over the last years, the use of dynamic (reversible) covalent and supramolecular polymer networks has emerged as an alternative option for the SMP design due to their triggable and reversible nature.^8^ Indeed, the ability to form, to break and to subsequently reform a specific bond or interaction upon an external stimulus has appeared as a great opportunity to tune SMP architectures. The presence of reversible binding groups in SMPs ensures the stabilization of the temporary shapes, imparts recyclability properties, enables alternative non-thermal triggers for the switching mechanism or engenders extra functionality like self-healing.^9^ Whether these groups are covalent or supramolecular in nature, they can serve as either permanent or temporary net-points. One of the most illustrating dynamic covalent groups used in SMP design are the thermoreversible Diels–Alder (DA) adducts, *e.g.*, the furan-maleimide DA adducts. While the [4+2] cycloaddition proceeds at room temperature, the reverse reaction (retro-DA) becomes generally favorable above 100 °C. The shape reprogramming of some SMPs based on biodegradable polymers such as poly(ε-caprolactone) (PCL) and poly(lactide) (PLA) as the switching domain was allowed by the presence of such DA adducts as widely reported in the literature.^5,10–16^ Simultaneously, relevant non-covalent interactions such as hydrogen bonding,^17–20^ metal–ligand coordination,^8,21^ ionic interactions^22–24^ or π–π stacking^25^ were integrated into SMP designing. Among this wide variety of supramolecular interactions, the hydrogen bonds have acquired a specific attention to build reversible polymeric materials, being able to display SMP properties.^17,26,27^ In 1997, Meijer and co-workers revealed that the self-complementarity hydrogen bonding arrays of 2-ureido-4-pyrimidone (UPy) end groups in linear polymers allowed obtaining thermodynamically controlled self-assembling architectures.^28^ Thanks to the strong dimerization of the UPy units (dimerization constant greater than 10^6-7^ M^−1^ in chloroform) and a predictable tautomerization,^29–32^ polymers displaying a high degree of polymerization could be easily obtained.^33,34^ In addition, the UPy functional groups are also present as dimers in the solid state *via* a donor–donor–acceptor–acceptor (DDAA) array.^32^ In this context, the quadruple hydrogen bonding motifs of UPy molecules have been then employed to design SMPs.^9^ As the hydrogen bonding is highly-reversible and strongly temperature dependent,^35^ UPy groups were first used as molecular switches^36^ exhibiting excellent shape memory effect (SME), related to the temperature dependence of the hydrogen bonding dynamics. Interestingly, the presence of UPy groups as pendant groups in a chemically cross-linked acrylic copolymer afforded the material to change shapes twice and to fix two metastable temporary shapes. The triple-shape capability resulted from the polymer *T*_g_ and the dissociation of self-complementarity hydrogen as the two molecular switches.^37^ Using novel approaches, it was also demonstrated that UPy moieties could be imparted into SMPs to support the stabilization of the permanent shape. Interestingly, Xiao *et al.* developed a one-step procedure to create UPy-functionalized poly(ε-caprolactone)/poly(*para*-dioxanone) (PPDO) interpenetrated polymer networks (IPNs) with triple shape ability. A covalent cross-linking was established between PPDO and hexamethylene diisocyanate and a supramolecular network was formed through self-complementarity hydrogen bonds between the UPy end-groups of the star-shaped PCL.^38^ A PCL–PPDO covalently cross-linked network was prepared for comparison. In the new covalent IPN network, the ability of each polymer segment to achieve crystalline phase was greater compared to this star-shape covalently PCL–PPDO network and the resulting t-SME was excellent. In a further work, a dynamic network was produced *via* the self-complementarity quadruple hydrogen bonding through the UPy–poly(tetramethylene ether)glycol and 4-UPy star-shaped PCL.^39^ Besides the t-SME ensured by the presence of two separated *T*_m_ and the UPy dimer net-points, the material exhibited a good self-healing feature due to the reversibility of the dynamic network. Recently, supramolecular poly(vinyl alcohol) (PVA) networks were prepared through the dimerization of UPy motifs into the macromolecular chains by Chen *et al.*^40^ In this particular work, the PVA chains acted as thermo-reversible phase, whereas the presence of UPy dimers formed “cross-linked” net-points, ensuring the fixation of the permanent shape. In addition to the direct thermally triggered recovery, water-induced recovery was observed when the samples were immersed because of the plasticizing effect of the water molecules decreasing the polymer *T*_g_. Thereby, it was proven that conventional chemical covalent cross-linked bonds might be potentially replaced by quadruple hydrogen bonding of UPy arrays to easily obtained functional materials.

In the present work, we herein describe the design of new supramolecular shape-memory polymeric networks made of semi-crystalline PCL oligoester segments, as switching domains, and UPy unit pendant side-groups, as permanent domain. In contrast to Chen *et al.*,^40^ we report a flexible pathway to readily incorporate UPy units with hydroxyl functionalities (UPy(OH)_2_) in α,ω-dihydroxy PCL-oligomers through coupling reactions. Indeed, due to their biodegradability, biocompatibility and low melting temperature, PCL oligomers indeed represent promising candidates for new bio-applicative SMPs.^41^ Practically, the synthesis of UPy(OH)_2_ was first developed and following polyurethanes (PUs) from commercial oligoPCL and UPy(OH)_2_ were obtained by chain-extension reaction in dimethylformamide (DMF) at 80 °C in the presence of hexamethylene diisocyanate (HMDI) as coupling agent (1.1 equivalents relative to OH) and dibutyltin dilaurate (DBTL) as catalyst. To control the density of supramolecular net-points, and therefore of the fixing domain, cellulose nanocrystals (CNCs) were added into the PUs networks. Interestingly, cellulose nanocrystals have been considered as a compatible reinforcing nanomaterial for diverse biopolymers.^42^ They are abundantly produced from renewable resources and offer several advantages such as stiffness, biocompatibility, low density, low cost, high aspect ratio.^43^ High modulus and interconnected CNCs networks could be formed in polymer matrix containing polar group or hydroxyl end-functionality (*e.g.*, PLA, PEG) *via* hydrogen bonding due to the presence of abundant hydroxyl groups on their surface.^3^ Moreover, UPy-functionalized CNCs are utilized as fillers in a UPy-modified telechelic building block for the design of light-healable supramolecular nanocomposites.^44,45^ In the present study, the advantage is taken from the presence of free hydroxy groups on the neat (unmodified) CNCs surface and their interactions with UPy in order to finely dispersed them into PU materials as to reinforce the supramolecular networks with additional hydrogen bondings.

## (2) Experimental part

### (2.1) Materials

Ethyl acetoacetate (EAA, AR grade, Kelong Reagent Corp.), guanidine carbonate (AR grade, Sigma Aldrich), 1,6-hexamethylene diisocyanate (HMDI, >98.0%, TCI) and 2-amino-2-methyl-1,3-propanediol (AMPD, 99%, Acros) were used without further purification. Ethanol (AR grade) and chloroform (AR grade) were purchased from Kelong Reagent Corp. Chloroform was dried with molecular sieve (3A) before use. α,ω-dihydroxyl poly(ε-caprolactone) (PCL(OH)_2_, Capa™ 2402, *M*_n_ = 4000 g mol^−1^, MWD = 1.48) was used as kindly supplied by Perstorp. Dibutyltin dilaurate (DBTL, Alfa Aesar, >98%) and dimethylformamide (99.8%, extra dry over molecular sieve, Acros) were used as received. Pure ramie fibers were obtained from Stucken Melchers GmbH & Co. (Germany).

### (2.2) Preparation of 1-(6-(3-(1,3-dihydroxy-2-methylpropan-2-yl)ureido)hexyl)-3-(6-methyl-4-oxo-1,4-dihydropyrimidin-2-yl)urea (UPy(OH)_2_)

UPy(OH)_2_ was obtained after three successive reaction steps. The two first intermediate products were obtained as reported earlier.^39^ First, a suspension of guanidine carbonate (25.48 g, 0.198 mol) and EAA (16.2 g, 0.18 mol) in ethanol (200 mL) was refluxed for 8 h. The resulting white mixture was filtered on a Buchner funnel and then washed in ethanol, deionized water and acetone, successively. 6-Methylisocytosine (MIC) was finally obtained after further drying in vacuum oven overnight (yield = 74%). In the second step, MIC (15.0 g, 0.12 mol) was dissolved in HMDI (143 mL, 0.888 mol) under nitrogen flow and the reaction was conducted at 100 °C for 12 h. After cooling, the mixture got precipitated and washed with petroleum ether and then dried under vacuum. 1-(6-Isocyanate)-3-(6-methyl-4-oxo-1,4-dihydropyrimidin-2-yl)urea (UPy-NCO) was recovered with high yield (98%). Finally, UPy-NCO (15.0 g, 51.2 mmol), AMPD (8.06 g, 76.8 mmol) and 450 mL of dry chloroform were added into a round-bottom flask under nitrogen atmosphere and heated at 60 °C for 6 h. After filtration of the mixture on a Buchner funnel, the recovered white powder was then dissolved into 240 mL of DMF. A following centrifugation step eliminated the by-products and the supernatant was poured into 1.5 L of ether absolute. The precipitate was recovered by filtration under vacuum. After washing with acetone, UPy(OH)_2_ was dried overnight at 40 °C with a yield of 86%. ^1^H NMR spectra in DMSO-d_6_ of MIC, UPy-NCO and UPy(OH)_2_ are showed in ESI (see Fig. S1, S2 and S3,† respectively).

### (2.3) Synthesis of PUs

PCL(OH)_2_ and UPy(OH)_2_ were first introduced in a previously flame-dried round-bottom flask and dried 1 h under vacuum. Then, between 20 and 25 mL of anhydrous DMF were added under nitrogen flow. The solution was heated up to 60 °C until its complete dissolution. HMDI and DTBL were successively introduced under nitrogen flow. All syntheses were conducted at 90 °C under nitrogen flow for 6 h. After reaction, mixtures were injected into a horizontal Teflon mold in a glass autoclave to evaporate the solvent for 24 h. The resultant films were first transferred into a drying oven overnight at 60 °C and put under vacuum for 24 h at 40 °C.

### (2.4) Preparation of cellulose nanocrystals (CNCs)

CNCs were prepared from ramie fibers as reported in the literature.^46^ Briefly, 60 g of purified ramie fibers were cut into small pieces and treated with 1000 mL of a 4% NaOH solution at 80 °C for 2 h to remove residual contaminants. The fibers were then submitted to acid-hydrolysis with 800 mL of sulfuric acid solution (65% wt) at 55 °C for 30 min and under continuous mechanical stirring. The obtained suspension was filtered through a sintered glass no. 1 to remove macroscopic fragments from unhydrolyzed fibers. The suspension was thoroughly washed with water by centrifugation and dialyzed against deionized water until neutrality. The suspension was concentrated to 4% wt and constituted the stock suspension. The resulting CNCs display an average length around 200 nm and an average width of 7–10 nm according to TEM analyses.

#### Dispersion of CNC in DMF

An aqueous suspension of CNCs was solvent-exchanged from water to DMF. 10 g of DMF was added to 10 g of the aqueous dispersion of CNCs, then the mixture was transferred to a 100 mL flask and water was evaporated using rotavapor. Finally, the suspension was ultrasonicated for 5 min to re-disperse the CNCs. The concentration of the final suspension was determined gravimetrically to be 3.7% wt.

### (2.5) Preparation of nanocomposites

First, the suspension of CNCs in DMF was ultrasonicated for 10 minutes. Meanwhile, a specified amount of PU was solubilized in 10 mL of DMF. Then, both mixtures were put together and mixed overnight. Solution was ultrasonicated for 15 minutes. Films containing 2.5, 5, 10 and 15 wt% of nanoparticles were obtained by casting the mixtures in a Teflon mold and drying in a glass autoclave at 80 °C for 24 h. Resulting films were placed in a vacuum oven at 40 °C for a few hours. To carry out DMTA analyses and to homogenize the thickness, films were hot-pressed at 110 °C.

### (2.6) Characterization techniques

^1^H NMR spectra were recorded with Bruker AV400 spectrometers (Bruker, Germany) at 400 MHz in DMSO-d_6_ and CDCl_3_ using TMS as an internal reference. Size-exclusion chromatography was performed on a Waters apparatus equipped with a model 1515 pump, a Waters model 717 autosampler and a 2414 refractive index detector, using monodispersed polystyrene standards to get a calibration curve. The elution solvent was chloroform at a flow rate of 1.0 mL min^−1^ at 30 °C. The sample concentration was about 2.5 mg mL^−1^. ATR Fourier-transform infrared (FTIR) spectra were recorded using Bruker Tensor 17 spectrometer. Thermal properties of precursors and PUs were determined by DSC measurements using a DSC Q200 from TA Instruments under an ultra-high purity nitrogen flow. The following “heat/cool/heat” procedure was applied to all samples: first heating to 200 °C (10 °C min^−1^) in order to erase the thermal history of samples, cooling to −80 °C (10 °C min^−1^), followed by a second heating to 200 °C (10 °C min^−1^). Sample weights were generally in the range of 5–10 mg. The thermomechanical properties of the PUs and their nanocomposites were tested with a DMA Q800 apparatus from TA Instruments in a multi-frequency strain mode (tension mode). Samples were heated at 3 °C min^−1^ from −80 to 70 °C at a frequency of 1 Hz with an oscillation amplitude of 10 μm and a force track of 125%. The morphology of the samples was observed using a scanning electron microscope (SEM) (JSM-5900LV, Jeol Co., JP). The observed cross-section was prepared by fracturing the samples in liquid nitrogen. The accelerating voltage was 20.0 kV and magnification level was comprised between 2000 and 80 000×. Rheological measurements were performed using a ARES Rheometer from Rheometrics. Cone and plate geometry (diameter 25 mm) was used to register the viscoelastic response. Strain sweep measurements were first performed at 110 °C with a frequency of 1 Hz. Then, frequency sweep measurements were recorded in linear regime conditions at a temperature of 110 °C with a strain of 10%. One-way shape-memory properties were determined using the Q800 apparatus in stress-controlled mode on rectangular specimens of approximately 15 mm × 5 mm × 0.30 mm cut from hot-pressed films. Note that for each sample, a heating ramp (5 °C min^−1^) and a cooling ramp (3 °C min^−1^) were successively applied in order to limit the variation of strain at high temperature. The typical four-step program was applied to determine the one-way behavior: (a) apply of a given load at a temperature higher than the second *T*_m_ of PU system (*T* = 100 °C) to stretch the sample; (b) cooling in two steps to 60 °C and then to 0 °C (10 min-isotherm) at 2 °C min^−1^ to fix the temporary shape; (c) quick unloading; (d) final heating to 95 °C at 3 °C min^−1^ followed by a 10 min isotherm to let the sample recovered to its initial shape. The one-way SME was characterized by the shape fixity ratio *R*_f_, representing the ability to maintain temporary shape and by shape recovery ratio *R*_r_ which shows the extent of the recovery, defined as followed:
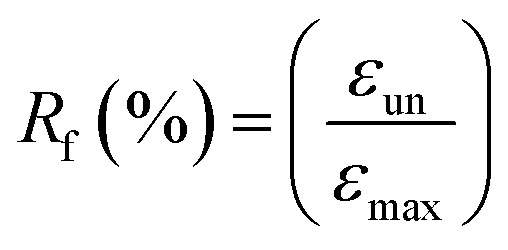

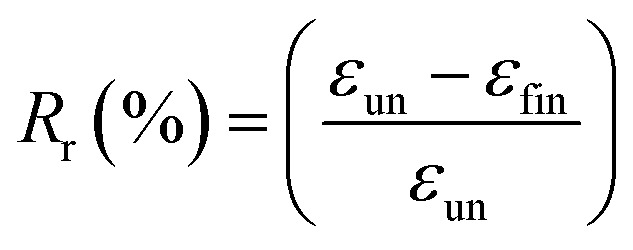
where *ε*_un_ is the strain after cooling and unloading, *ε*_max_ is the strain obtained before the constant loading was released and *ε*_fin_ is the strain obtained after heating in the step of recovery.

## (3) Results and discussions

### (3.1) Synthesis and characterization of PCL–UPy polyurethanes

**Scheme 1:**
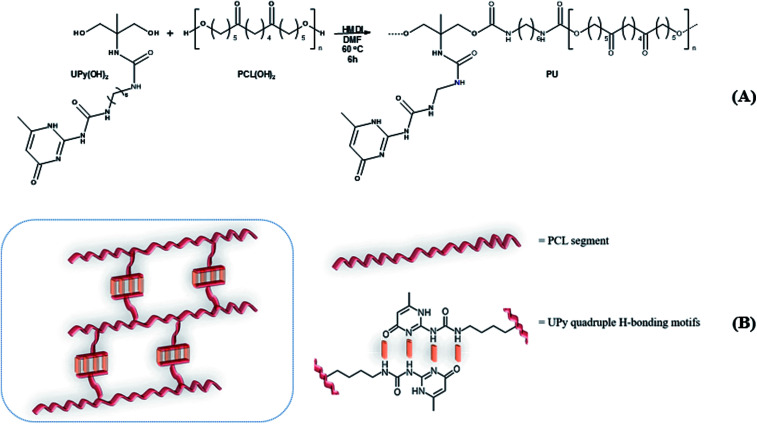
Synthetic pathway to (A) PUs and (B) schematic of their supramolecular network.

Three PCL-based PUs with different UPy pendant motifs (30, 50 and 70 mol% according to Table 1) were obtained *via* a chain-extension reaction between commercial α,ω-diol PCL oligomer, UPy-diol monomer and 1,6-hexamethylene diisocyanate (HMDI) as coupling agent performed in dry DMF (Scheme 1(A)). Solution version of the coupling reaction was here preferred to reactive extrusion in order to maintain linearity of the polyurethane chains and avoid undesirable side reactions such as any crosslinking.

**Table 1:** Composition, molecular and thermal characteristics of PUs

Sample identification	PCL(OH)_2_/UPy(OH)_2_ [mol mol^−1^%]	Conv.^a^ [%]	*M*_n_ b (PS) [g mol^−1^]	*Đ* b	*T*_m_ c [°C]	Δ*H*_m_^c^ [J g^−1^]
PU (1)	50/50	100	30 000	7	47	32.09
PU (2)	70/30	100	35 000	4.4	52	53.50
PU (3)	30/70	100	15 000	1.5	44	33.69

a Conversion as determined by FT-IR measurements.

b Experimental number-average molar mass (*M*_n_) and dispersity (*Đ*) as determined by SEC (CHCl_3_) upon PS calibration.

c Melting temperature as determined by DSC measurements and melting enthalpy as recalculated according to solely PCL content (from −80 °C to 100 °C at a heating rate of 10 °C min^−1^; second heating cycle).

**Fig. 1:**
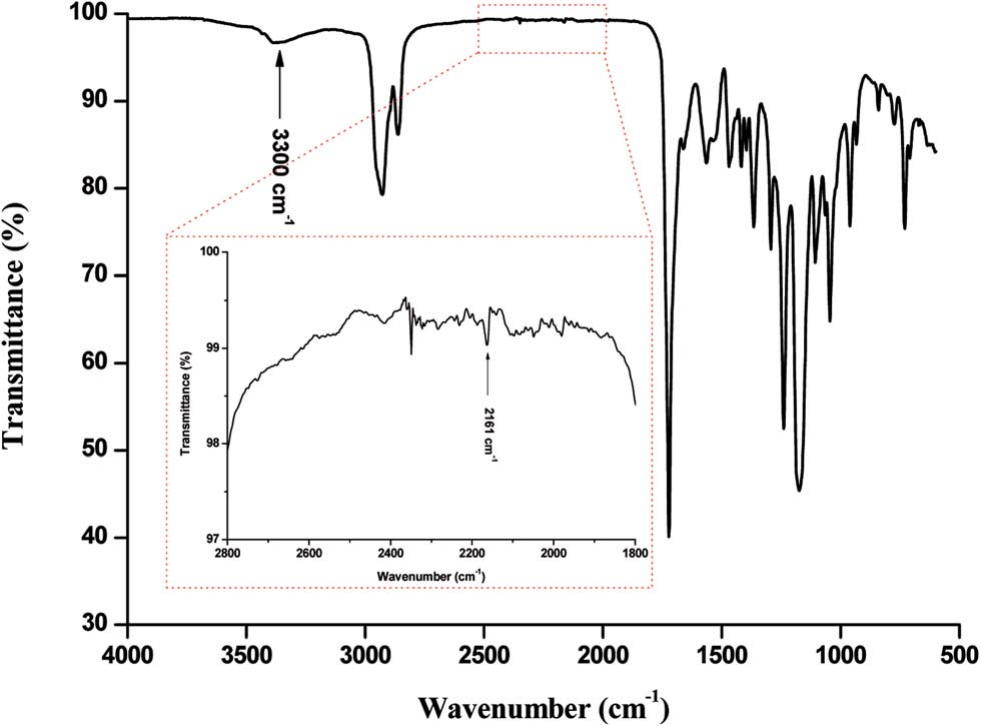
FTIR spectra of PU (1).

**Fig. 2:**
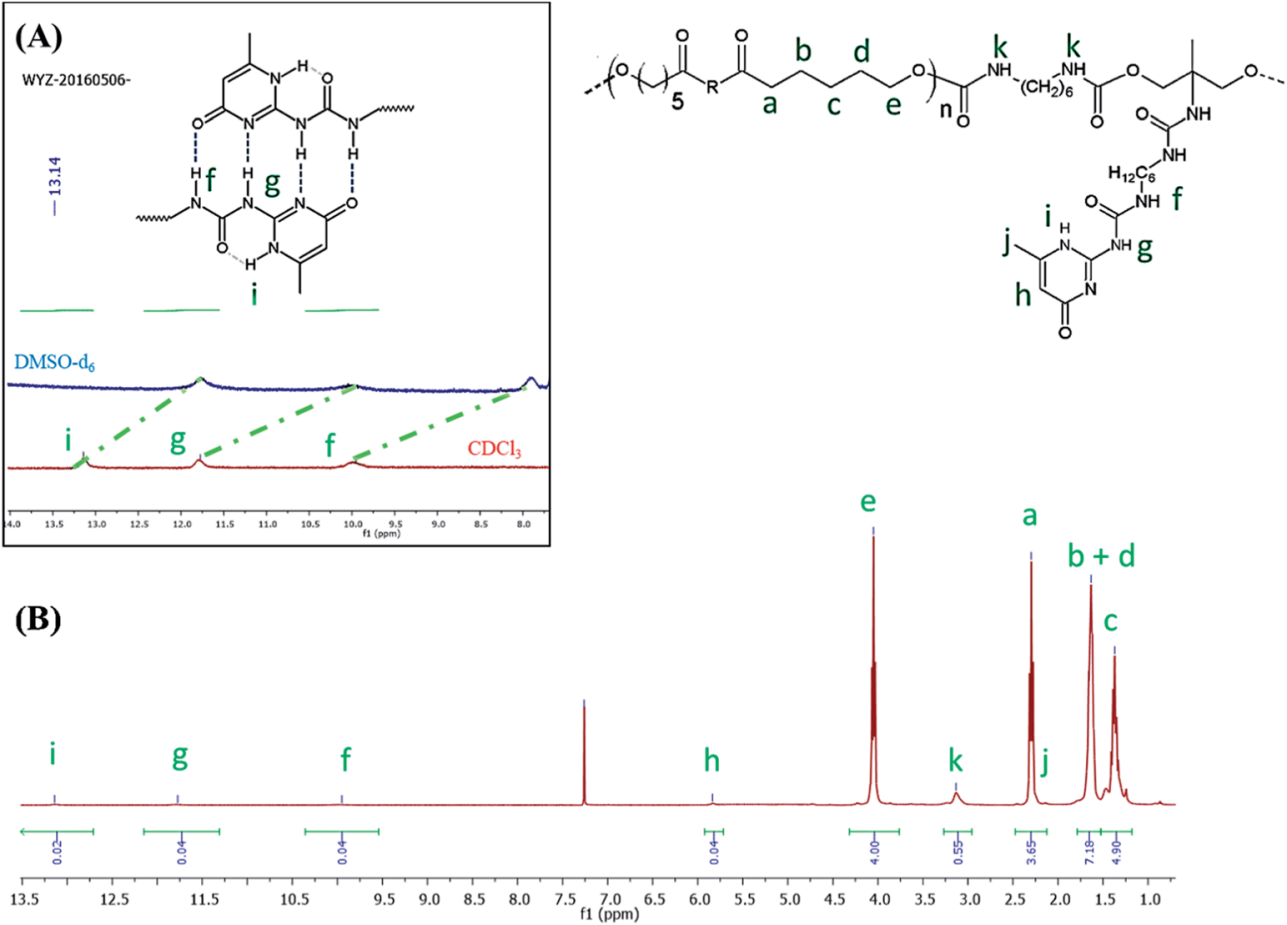
^1^H NMR spectrum of PU (1) in CDCl_3_ (A) and in DMSO-d_6_ ((B), blue spectrum).

FTIR analysis of the products (Fig. 1) presented barely visible band around 2200–2300 cm^−1^ assigned to unreacted isocyanate (–N=C=O) functionality and a large band around 3300 cm^−1^ ascribed to the presence of hydrogen bonds, mainly originating from dimerization of UPy moieties (*i.e.*, four hydrogen bonds per dimer, Scheme 1(B)).^47^ However, hydrogen bonding between PUs chains or PUs and UPy (carbonyl group of urethane bond or the ester moieties along the PCL backbone and –NH moieties) cannot be neglected. These hydrogen bonds usually trigger a poor solubility of PUs materials in common solvents^48^ Interestingly, the UPy enriched PUs synthesized herein were readily soluble in chloroform upon a vigorous stirring for one hour, thus allowing to monitor the chain-extension reaction by SEC and ^1^H NMR. Though the molecular parameters recorded by SEC must be considered with precaution, it comes out that *M*_n_ of the different PUs increases with PCL content (Table 1), proving that chain-extension is taking place. In addition, the ^1^H NMR spectrum of PU (1) as illustrating example reveals the absence of the peak at 3.65 ppm respective to end-chain group of PCL (–CH_2_–OH), and shows down field signals at *δ* = 13.14, 11.77 and 9.95 ppm (Fig. 2(A)), assigned to UPy-based intermolecular hydrogen bonds.^30^ In comparison, chemical shifts for polyester–polyurethane H-bonds, expected to occur at *δ* = 9.95, 9.76, 9.68 and 8.66 ppm (ref. 45) are not observed.

The presence of a pyrimidyl resonance peak at 5.84 ppm (proton “h”) showed that the UPy dimers adopted keto-tautomeric form within the PU structure.^30^^1^H NMR of PU (1) was also recorded in DMSO-d_6_. This strong hydrogen bond acceptor solvent disrupted the dimerization of the UPy groups and the NH signals shifted to *δ* = 11.7, 10.2 and 7.7 ppm (blue spectrum in Fig. 2(B)). Thus, combining FTIR and ^1^H NMR measurements tends to confirm the presence of UPy inter-chain hydrogen bonds both in solvent like CHCl_3_ and in bulk state.

#### Thermal, mechanical, SME and recyclability investigations

Once the molecular structure of the PUs had been confirmed, their thermal behavior was studied by DSC and the thermal characteristics (melting temperature and melting enthalpy) are reported in the Table 1. As might be seen, a *T*_m_ ranging from 44 °C to 51 °C was recorded, indicating that the crystalline properties of the neat PCL were maintained in the design of supramolecular networks. The melting enthalpies (Δ*H*_m_) of PCL in PUs decreased with UPy molar content (from 53.50 J g^−1^ to 33.69 J g^−1^ for PU (2) and PU (3), respectively), suggesting that chain extension slows the crystallization of PUs, most probably due to hydrogen bonds formation, as shown earlier for poly(tetramethylene ether glycol)–UPy systems.^47^ Indeed, the dimerization of the pendant UPy groups might be expected to form a supramolecular network that would decrease the PCL chains mobility, restrict their arrangement at the juncture and thus lower the crystallization. This hypothesis was somehow supported by the thermo-mechanical properties and rheology evaluations (see ESI†).

**Fig. 3:**
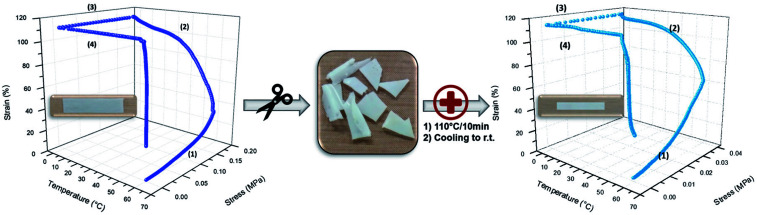
Recycling of PU (1): left – SME prior mechanical disintegration, right – SME after healing.

With this respect, relatively poor PU (3) and PU (2) mechanical resistance was clearly evidenced and ascribed to the lack of sufficient supramolecular network formation, related to the low amount of UPy functions present within these materials. Thus, only PU (1) was considered as a potential candidate for SMP (Fig. 3 left). As shown on the strain–stress–temperature curve, PU (1) was deformed to 110% of elongation by a constant load (*σ*_app_) of 0.18 MPa maintained during the cooling to 0 °C. After stress release, the temporary shape was fixed with a *R*_f_ value of 100%. After a second heating to 60 °C, the final shape was recovered to a residual elongation of 30% corresponding to a *R*_r_ of 73%. Like the PVA-based supramolecular network developed by Chen *et al.*,^40^ our PCL-based supramolecular network exhibits a good heating-responsive SME with an excellent *R*_f_ and suitable *R*_r_.

However, chemically cross-linked SMPs, even though displaying excellent SME, generally suffer from a lack of recyclability and/or processability. As in the case of PU (1) the SMP was crosslinked by hydrogen bonding with dynamics depending on temperature, recyclability of the material was expected. For confirming this hypothesis, PU (1) was chunked into pieces, and regenerated into a new film by compression molding at 110 °C for 10 minutes and SME was evaluated. Indeed, as shown in Fig. 3 (right), the new specimen also exhibited excellent shape-memory properties with a complete shape fixation (*R*_f_ = 100%) and a *R*_r_ value of 67%. Moreover, this behavior is repeatable, meaning that our supramolecular networks are easily re-processable and maintain SME properties.

### (3.2) PCL–UPy–CNC nanocomposites

**Fig. 4:**
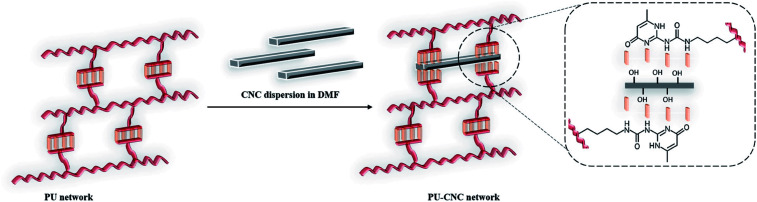
Schematic approach to design PCL–UPy–CNCs nanocomposites.

In an attempt to further improve thermal and mechanical properties of the supramolecular PU (1) network, CNCs were selected as suitable biocompatible nanofillers able to complement the network due the hydroxyl groups present on their surface (Fig. 4). Indeed, CNCs are known to promote microphase separation between soft and hard segments in PCL-based water borne polyurethanes and influence both glass transition temperature (*T*_g_) and crystallinity of the polyester.^49^ Moreover, the Young's modulus and the tensile strength increased with the CNC content due to the formation of three-dimensional network of hydrogen-bonding inter-actions between CNC particles and between CNC and WPU matrix.

Here below, PCL–UPy–CNCs nanocomposites were prepared by mixing in DMF solution the CNCs suspension and the previously solubilized PU (1), followed by a subsequent sonication. Nanocomposite films containing 2.5, 5, 10 and 15 wt% CNCs were then obtained by solvent casting.

#### CNC dispersability and PCL–UPy–CNC microstructure

**Fig. 5:**
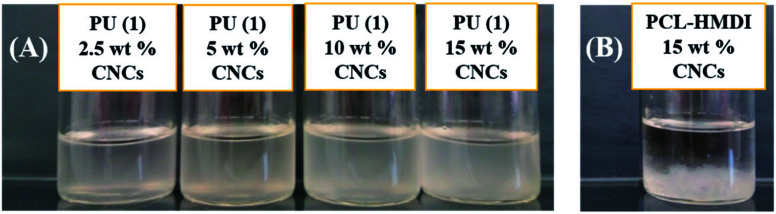
CNCs dispersion tests with the PCL–UPy–CNCs nanocomposites made from PU (1) (A), and PCL-based PU + 15 wt% CNCs nanocomposite (B) in chloroform.

In order to highlight the CNCs dispersion ability into the PCL hydrophobic matrix and the hydrogen bonding, a first visual dispersion test was carried out in CHCl_3_ as a bad dispersant for CNCs.^50^ For this, PCL–UPy–CNCs nanocomposites strips of approximatively 50 mg were swollen in 10 mL of CHCl_3_ at room temperature and upon a long-time vigorous mixing the stripes completely dissolved and the mixture became hazy (Fig. 5(A)). The resulting dispersions remained stable over a week, with no macroscopic CNCs aggregation and/or sedimentation. In comparison, a PU made only of PCL and HMDI and containing 15 wt% CNCs showed a dramatic aggregation of CNCs dispersion with complete CNCs sedimentation in just a few minutes (Fig. 5(B)), meaning that there were weak (or lacking) interactions between the CNCs hydroxyl groups and the urethane groups. Thereby, the presence of UPy side-groups enabled a fine dispersion of the nanofillers into hydrophobic PCL matrix as well as in chloroform, actually a very poor dispersant solvent for CNCs.

**Fig. 6:**
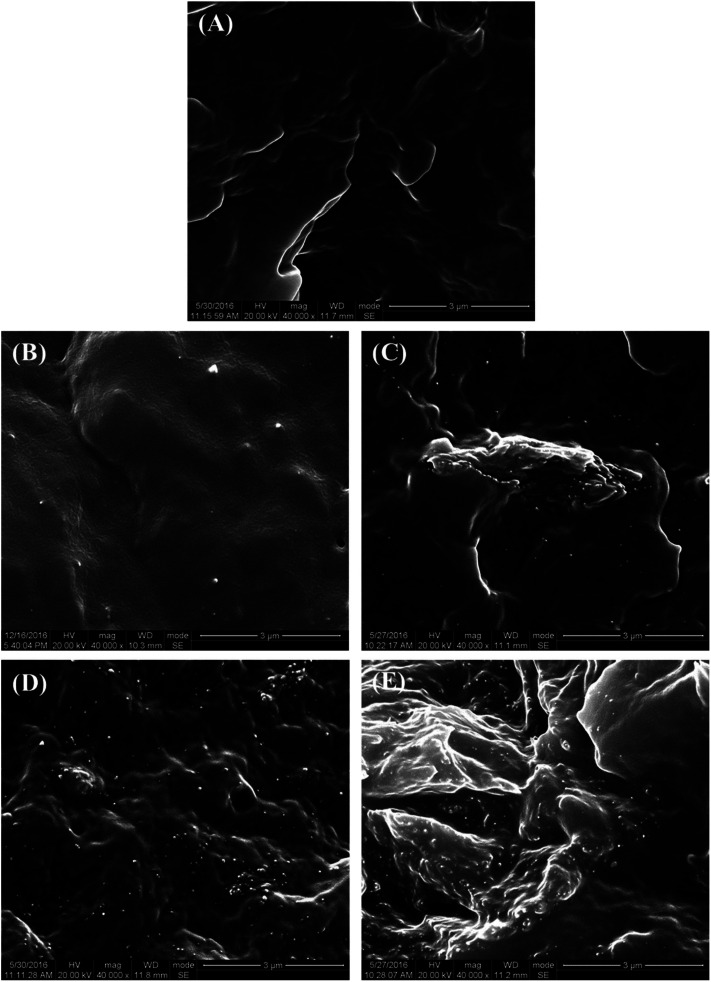
SEM images of the fractured surfaces of PU (1) based films reinforced with CNCs. (A) Neat PU (1), (B) PU (1)/CNCs 2.5 wt%, (C) PU (1)/CNCs 5 wt%, (D) PU (1)/CNCs 10 wt% and (E) PU (1)/CNCs 15 wt%.

The microstructure of the PCL–UPy–CNCs nanocomposites and PU (1) was then observed by SEM. Before, the samples were immersed into liquid nitrogen and cut off vertically to the fracture surface. Fig. 6 shows cross-sectional SEM images of the neat PU (1) (Fig. 6(A)) and nanocomposite films containing 2.5, 5, 10 and 15 wt% CNCs (Fig. 6(B)–(E)). As compared to the neat PU (1) film, CNCs were clearly identified in the matrix as white dots and proved located in the perpendicular plane of the film, as already reported in the literature.^51–54^ As the CNCs content increased, numerous white dots at the surface extended as well. At low nanofillers content, *i.e.* 2.5 and 5 wt%, a uniform distribution of the nanocrystals in the matrix with no large aggregates was observed, implying a good interaction between the CNCs and the UPy moieties. At 10 and 15 wt% of CNCs content, some voids appeared, suggesting CNCs aggregation. Nevertheless, together with the dispersion tests in chloroform, these images confirm the good dispersion ability of the CNCs into the PU (1) matrix.

#### Thermal, mechanical and SME investigations

The thermal properties of PCL–UPy–CNCs nanocomposites are listed in Table 2. Although *T*_m_ insignificantly varied with composition (for neat PU (1) see Table 1), Δ*H*_m_ increased with CNCs loading, and reached a maximum for PU (1) + 15 wt% CNCs suggesting a nucleation effect of well-dispersed CNC. However, these data are somehow deviating from the literature, where a maximum in enthalpy is often reported at 5 wt% nanofiller loading due to the formation of a well-developed cellulosic network and hindering of the polymer crystallites growth.^52^ One might suppose that CNC incorporation competes the UPy–UPy supramolecular network and allows UPy–CNC interactions, that preserve fine nanocrystals dispersion even at higher filler loadings of 15 wt% and allow their nucleation effect to be further expressed.

**Table 2:** Thermal and shape-memory properties of PU (1) + CNC nanocomposites

Sample identification	*T*_m_ a [°C]	Δ*H*_m_^a^ [J g^−1^]	*σ*_app_ (MPa)	*R*_f_ [%]	*R*_r_ [%]
PU (1) + 2.5 wt% CNCs	50	37.44	0.25	∼100	100
PU (1) + 5 wt% CNCs	49	36.41	0.18	∼100	96
PU (1) + 10 wt% CNCs	49	40.16	0.28	∼100	71
PU (1) + 15 wt% CNCs	49	41.76	0.38	∼100	76

a Melting temperature as determined by DSC measurements and melting enthalpy as recalculated accounting for solely PCL content (from −80 °C to 100 °C at a heating rate of 10 °C min^−1^; second heating cycle).

**Fig. 7:**
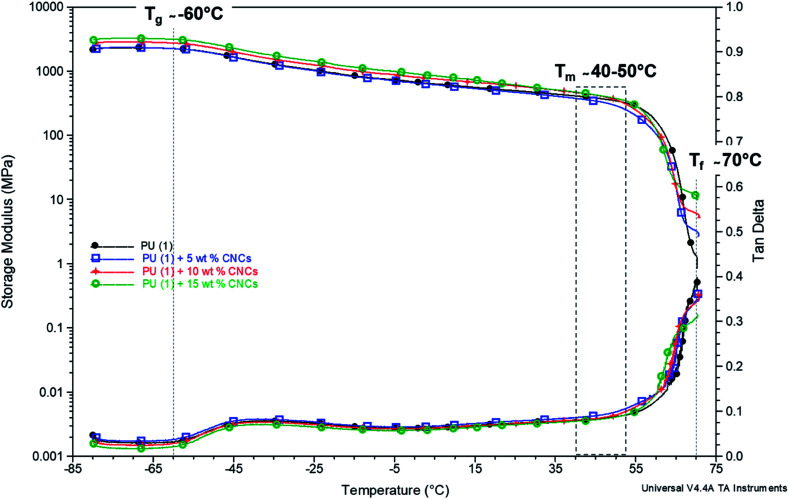
Storage modulus and tan *δ* – temperature dependence of PU (1) with CNCs content increasing from 0 to 10 wt% (the curve for CNC loading of 2.5 wt% is not shown as it overlays with 0 and 5 wt%).

The results are somehow confirmed by the thermomechanical properties of the composites (Fig. 7). Indeed, *T*_α_ (about −60 °C) and *T*_m_ (between 40 and 50 °C) revealed in good agreement with DSC data. Interestingly, a second rubbery plateau occurred above *T*_m_ at 70 °C. This phenomenon was already reported for other PU-systems and ascribed to the formation of supramolecular structures by the aggregation of dimerized groups.^47^ Only after, complete flowing of the system occurred and the temperature at which this last phenomenon occurs is called “flowing temperature” (*T*_f_). In the case of PU (1) + CNC nanocomposites, the *T*_f_ (defined as the end of the second rubbery plateau) as recorded from the DMTA curves was situated at about 70 °C, thus identifying it as an upper service temperature (Fig. 7). The value of the storage modulus at this point increased upon increasing the CNC loading and reached 1, 3, 5 and 10 MPa, for CNC content of 0, 5, 10 and 15 wt%, respectively, thus confirming that CNCs act as reinforcing agents in the supramolecular network of PCL–UPy polyurethane chains.

**Fig. 8:**
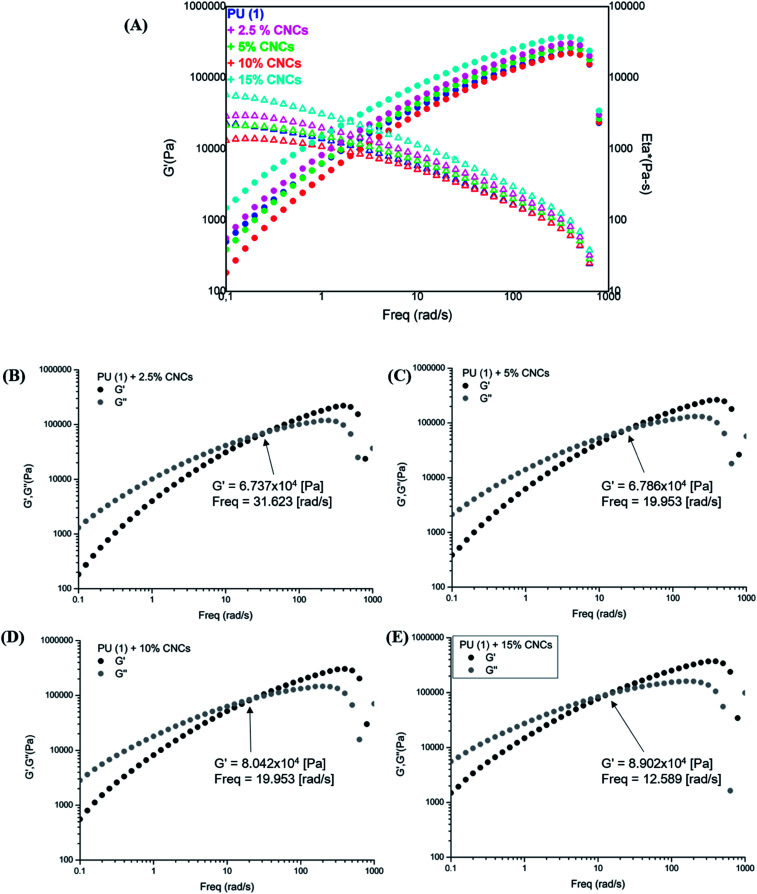
Frequency sweep tests for PU (1) and its nanocomposites: (A) *G*′ and Eta* profiles, (B)–(E) *G*′ and *G*′′ for 2.5, 5, 10 and 15 wt% CNCs content, respectively.

The rheological characterization of the nanocomposites (Fig. 8) shows their elastic modulus and the complex viscosity evolution in function of the frequency. As one can observe, samples with low CNCs content, *i.e.*, 2.5 and 5 wt%, displayed a slightly reduced viscosity with respect to the neat PU (1), most probably due to the involvement of some UPy groups in hydrogen bonding with CNCs. However, composites containing 10 and 15 wt% CNC presented higher *G*′ and viscosity in the low frequency range as expected from reinforcement. Interestingly, storage and loss modulus indicated a liquid-like behavior (*G*′′ > *G*′) in the low frequency range (Fig. 8(B)–(E)), independently on CNC loading. A clearly marked cross-over of *G*′ with *G*′′ appeared at 32 rad s^−1^ for the PU (1) + 2.5 wt% CNC which was twice higher the value recorded for the neat PU (1) (Fig. SI2†). Thus, disrupted UPy–UPy interactions and formation of new UPy–CNC network are confirmed. It might be assumed, that the CNC loading is yet too low for sufficient network stabilization. Indeed, upon increasing the CNC content, this cross-over shifted to 19 rad s^−1^ (PU (1) + 5 wt% CNC) and reached 12.6 rad s^−1^ for PU (1) + 15 wt% CNC, confirming the stabilization of UPy–CNC network and fixing the percolation at about 5 wt% nanofiller loading. As this behavior certainly originates from the different affinity between CNCs–CNCs, CNCs–UPy and UPy–UPy groups, it might be suggested that dimerization between UPy groups is enthalpically less favorable than the formation of clusters CNC–UPy or CNC–CNC in the supramolecular network.

Concerning the SME of the PU (1) + CNC nanocomposites (Table 2), a *R*_f_ value of 100% and a *R*_r_ value of 96% were obtained for 5 wt% CNCs sample, while both *R*_f_ and *R*_r_ values were close to 100% at 2.5 wt% CNCs content. This suggests that at low CNC loading (best illustrated with the case of PU (1) + 2.5 wt% CNC) some involvement of the UPy groups in hydrogen bonds with CNCs occurs, although their amount is yet not sufficient for a stable percolated network with restricted mobility to be formed. Instead, stabilization and higher mobility of the chains in the supramolecular network are ensured and best shape-memory effect is observed. On the contrary, nanocomposites with higher CNCs contents (10 and 15 wt%) did not exhibit improved shape-memory features, keeping recovery behavior similar to the neat PU (1). For these samples, the percolation threshold between CNCs was quickly reached, thus modifying the SME properties of the materials. Accordingly, nanocomposites containing 2.5 and 5 wt% CNCs seemed to propose a good compromise in terms of shape-memory properties.

## Conclusions

PCL–UPy linear polyurethanes with UPy content from 30 to 70 mol% were successfully obtained *via* chain-extension reaction with diisocyanates. For this purpose, the synthesis of UPy-diol was first developed. FTIR and ^1^H NMR were used to confirm chemical structure and composition of all molecules, and SEC in chloroform attested the targeted increase in molar mass for the PUs. Thermal properties evaluation showed decreased melting enthalpy upon increasing UPy content and suggested that UPy dimerization forms a supramolecular network that might restricts the mobility of the PCL chains and thus reduce polyester crystallinity. The hypothesis was further confirmed by mechanical and rheology tests drawing PU (1) as most appropriate candidate for SMP.

The formation of a reversible supramolecular network based on hydrogen bonding was confirmed to be a good prerequisite for SMP recycling together with SME preservation.

Further improvement in PU (1) properties was achieved by finely dispersing CNCs as biobased nanofillers able to form hydrogen bonds. The presence of interactions between UPy moieties and CNCs in the nanocomposites was demonstrated by dispersion tests and SEM. Evaluations of the influence of CNCs loading by DSC, DMTA and rheology attested the presence of CNC–UPy interactions competing UPy–UPy dimerization; and tests on SME revealed improved *R*_r_ values for PU (1) nanocomposites of low CNC loading (2.5 and 5 wt%).

The here obtained nanomaterials, combining PCL biocompatibility with CNCs bio-based origin and biocompatibility might find possible applications in areas where both SMP and biocompatibility are needed for biomedical applications.

## Conflicts of interest

There are no conflicts to declare.

## Supplementary Material

RA-008-C8RA03832E-s001

## References

[cit1] Sun L., Huang W., Ding Z., Zhao Y., Wang C., Purnawali H., Tang C. (2012). Mater. Des..

[cit2] Liu T., Zhou T., Yao Y., Zhang F., Liu L., Liu Y., Leng J. (2017). Composites, Part A.

[cit3] Pilate F., Toncheva A., Dubois P., Raquez J.-M. (2016). Eur. Polym. J..

[cit4] Qin H., Mather P. T. (2009). Macromolecules.

[cit5] Raquez J.-M., Vanderstappen S., Meyer F., Verge P., Alexandre M., Thomassin J.-M., Jérôme C., Dubois P. (2011). Chem.–Eur. J..

[cit6] Peacock A. J. (2001). J. Macromol. Sci., Part C: Polym. Rev..

[cit7] Chodák I. (1998). Prog. Polym. Sci..

[cit8] Zhang T., Wen Z., Hui Y., Yang M., Yang K., Zhou Q., Wang Y. (2015). Polym. Chem..

[cit9] Lewis C. L., Dell E. M. (2016). J. Polym. Sci., Part B: Polym. Phys..

[cit10] Inoue K., Yamashiro M., Iji M. (2009). J. Appl. Polym. Sci..

[cit11] Djidi D., Mignard N., Taha M. (2015). Ind. Crops Prod..

[cit12] Rivero G., Nguyen L.-T. T., Hillewaere X. K. D., Du Prez F. E. (2014). Macromolecules.

[cit13] Defize T., Thomassin J.-M., Alexandre M., Gilbert B., Riva R., Jérôme C. (2016). Polymer.

[cit14] Defize T., Riva R., Raquez J. M., Dubois P., Jérôme C., Alexandre M. (2011). Macromol. Rapid Commun..

[cit15] Defize T. , Riva R., Thomassin J. M., Jérôme C. and Alexandre M., 2011

[cit16] Nguyen L.-T. T., Truong T. T., Nguyen H. T., Le L., Nguyen V. Q., Van Le T., Luu A. T. (2015). Polym. Chem..

[cit17] Nair K. P., Breedveld V., Weck M. (2008). Macromolecules.

[cit18] Chen H., Liu Y., Gong T., Wang L., Zhao K., Zhou S. (2013). RSC Adv..

[cit19] Peterson G. I., Dobrynin A. V., Becker M. L. (2016). ACS Macro Lett..

[cit20] Ragin Ramdas M., Reghunadhan Nair C. P., Santhosh Kumar K. S. (2017). Eur. Polym. J..

[cit21] Xie F., Huang C., Wang F., Huang L., Weiss R. A., Leng J., Liu Y. (2016). Macromolecules.

[cit22] Dong J., Weiss R. (2013). Macromol. Chem. Phys..

[cit23] Raidt T., Hoeher R., Meuris M., Katzenberg F., Tiller J. C. (2016). Macromolecules.

[cit24] Odent J., Raquez J.-M., Samuel C., Barrau S., Enotiadis A., Dubois P., Giannelis E. P. (2017). Macromolecules.

[cit25] Wang Q., Bai Y., Chen Y., Ju J., Zheng F., Wang T. (2015). J. Mater. Chem. A.

[cit26] Berl V., Schmutz M., Krische M. J., Khoury R. G., Lehn J. M. (2002). Chem.–Eur. J..

[cit27] Wang R., Xie T. (2010). Langmuir.

[cit28] Sijbesma R. P., Beijer F. H., Brunsveld L., Folmer B. J. B., Hirschberg J. H. K. K., Lange R. F. M., Lowe J. K. L., Meijer E. W. (1997). Science.

[cit29] Söntjens S. H., Sijbesma R. P., van Genderen M. H., Meijer E. (2000). J. Am. Chem. Soc..

[cit30] Hirschberg J. H. K. K., Beijer F. H., van Aert H. A., Magusin P. C. M. M., Sijbesma R. P., Meijer E. W. (1999). Macromolecules.

[cit31] Beijer F. H., Kooijman H., Spek A. L., Sijbesma R. P., Meijer E. (1998). Angew. Chem., Int. Ed..

[cit32] Beijer F. H., Sijbesma R. P., Kooijman H., Spek A. L., Meijer E. (1998). J. Am. Chem. Soc..

[cit33] Keizer H. M., Sijbesma R. P., Jansen J. F., Pasternack G., Meijer E. (2003). Macromolecules.

[cit34] Dankers P. Y., van Leeuwen E. N., van Gemert G. M., Spiering A., Harmsen M. C., Brouwer L. A., Janssen H. M., Bosman A. W., van Luyn M. J., Meijer E. (2006). Biomaterials.

[cit35] Folmer B. J., Sijbesma R., Versteegen R., Van der Rijt J., Meijer E. (2000). Adv. Mater. (Weinheim, Ger.).

[cit36] Li J., Lewis C. L., Chen D. L., Anthamatten M. (2011). Macromolecules.

[cit37] Ware T., Hearon K., Lonnecker A., Wooley K. L., Maitland D. J., Voit W. (2012). Macromolecules.

[cit38] Xiao L., Wei M., Zhan M., Zhang J., Xie H., Deng X., Yang K., Wang Y. (2014). Polym. Chem..

[cit39] Wei M., Zhan M., Yu D., Xie H., He M., Yang K., Wang Y. (2015). ACS Appl. Mater. Interfaces.

[cit40] Chen H., Li Y., Tao G., Wang L., Zhou S. (2016). Polym. Chem..

[cit41] Wang K., Strandman S., Zhu X. X. (2017). Front. Chem. Sci. Eng..

[cit42] Habibi Y., Lucia L. A., Rojas O. J. (2010). Chem. Rev..

[cit43] Mendez J., Annamalai P. K., Eichhorn S. J., Rusli R., Rowan S. J., Foster E. J., Weder C. (2011). Macromolecules.

[cit44] Biyani M. V., Foster E. J., Weder C. (2013). ACS Macro Lett..

[cit45] Çetin N. S., Tingaut P., Özmen N., Henry N., Harper D., Dadmun M., Sèbe G. (2009). Macromol. Biosci..

[cit46] Costes L., Laoutid F., Khelifa F., Rose G., Brohez S., Delvosalle C., Dubois P. (2016). Eur. Polym. J..

[cit47] Lu X., Wang Y., Wu X. (1992). Polymer.

[cit48] Zhu B., Feng Z., Zheng Z., Wang X. (2012). J. Appl. Polym. Sci..

[cit49] Cao X., Dong H., Li C. M. (2007). Biomacromolecules.

[cit50] Zhang X., Ma P., Zhang Y. (2016). Polym. Bull. (Berlin).

[cit51] Khelifa F., Habibi Y., Benard F., Dubois P. (2012). J. Mater. Chem..

[cit52] Benhamou K., Kaddami H., Magnin A., Dufresne A., Ahmad A. (2015). Carbohydr. Polym..

[cit53] Zhao Q., Sun G., Yan K., Zhou A., Chen Y. (2013). Carbohydr. Polym..

[cit54] Liu Y., Li Y., Yang G., Zheng X., Zhou S. (2015). ACS Appl. Mater. Interfaces.

