# Supplementary material for: Design of melt-recyclable poly(ε-caprolactone)-based supramolecular shape-memory nanocomposites
Source: RSC Adv. 2018 Jul 30;8(48):27119–30. doi: 10.1039/c8ra03832e (PMC9083248; doi:10.1039/c8ra03832e)
Supplement: RA-008-C8RA03832E-s001 [file RA-008-C8RA03832E-s001.pdf]

Electronic Supplementary Material (ESI) for RSC Advances.
This journal is © The Royal Society of Chemistry 2018

# Design of novel poly(ε-caprolactone)-based supramolecular shape-memory nanocomposites

Florence Pilate[1], Zhi-Bin Wen[2], Farid Khelifa[1], Yan Hui[2], Sebastien Delpierre[1], Luo Dan[2], Rosica Mincheva[1], Philippe Dubois[1], Ke-Ke Yang[2], Jean-Marie Raquez[1,*]

SI

***(Thermo-)Mechanical properties***

It has been reported that the crystallization behavior of polymers connected with UPy end group via a urethane linker is influenced by the UPy stacking. The additional presence of urethane moieties stabilizes the stacks in the lateral direction via additional hydrogen bonding [66]. At contrast, bulky groups near to the UPy moieties can disrupt this crystallization. It was demonstrated that good mechanical properties of the formed network were dependent of the presence on the crystalline stacks. As shown in Table 1, PU (3) which presents the lower PCL-content, exhibits a low molecular weight, i.e. ~ 15 000 g $mol^{-1}$, meaning that its mechanical properties are most probably close to the oligo-PCL precursors, especially in terms of brittleness. Indeed, PU (3) based compressed films were very brittle, preventing any further characterizations. Therefore, only PU (1) and PU (2) were considered as candidates for supramolecular SMP design.

The thermo-mechanical behavior of their films (storage modulus (E') depending on temperature) was evaluated and shown in Figure SI1. As seen, a glass rubber transition above – 60°C relative to the $T_g$ of PCL, more pronounced in the case of PU (2) with the higher PCL-content, and a tensile storage modulus drop to a sloped plateau in the interval from -40°C to 40°C is observed. A second transition region close to the recorded $T_m$ is registered between 50°C and 60°C. For PU (1), an extended rubbery plateau followed this second transition to reach 0.3 MPa at 70°C. The formation of this last region was an indication of the successful network formation. Even though the PU (2) E' starts at higher values, it sharply decreased to 0.1 MPa during the second transition and plateau was almost inexistent. This suggests that the polymer structure was not sufficiently stabilized by the supramolecular UPy association (here considered as permanent domain), most probably due to the low UPy content.

DMTA in tensile mode testing was used to quantify the thermal actuation of PU (1) and (2). As already mentioned, after the first successive heating and cooling cycles erasing the "thermomechanical" history of the hot-pressed films, they were further heated to the deformation temperature (i.e. 60°C) to be stretched to a given elongation. However, PU (2) could not be correctly stretched as it brakes at 30-40% elongation independently on the applied deformation stress. It might be concluded that for this PU, the cohesion of the system during deformation above $T_m$ is not sufficient for ensuring formation of a supramolecular network. It might be suggested that the relatively low amount of UPy groups and urethane linkages, reduced the possibility of hydrogen bounding between UPy arrays or urethane bond and the PCL matrix. Consequently, only PU (1) was proved eligible as potential SMP candidate.

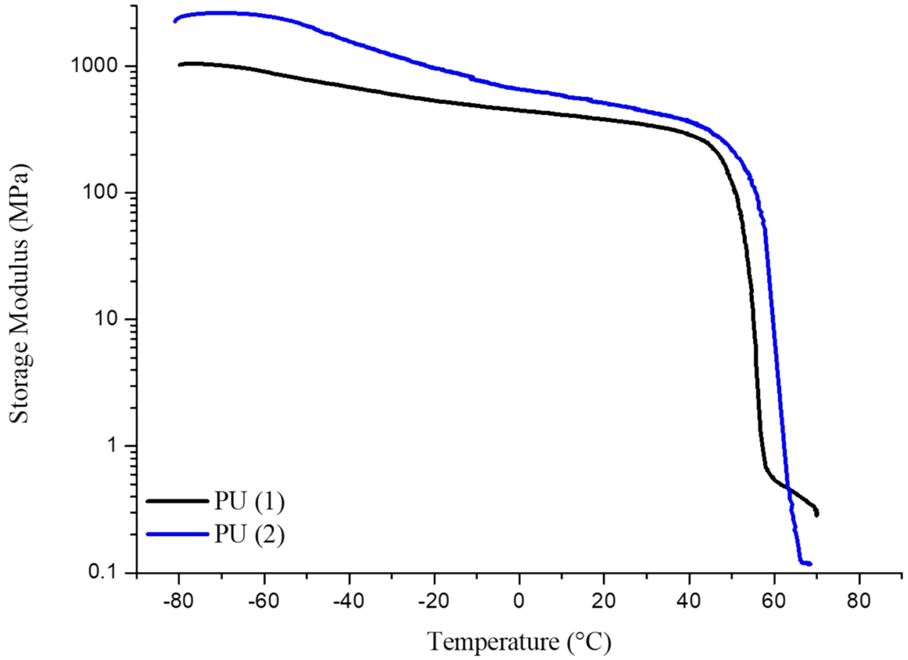


*Figure SI1. Storage modulus - temperature curves of PU (1) (black) and PU (2) (blue)*

### *Rheology*

These results were somehow confirmed by rheological measurements. The frequency sweep tests of PU (1), PU (2) and a commercially available PCL with a $M_n$ of about 50 000 g $mol^{-1}$ were recorded in their linear regime at 110°C. Considering Figure 4(a), one can conclude that PU (2) behaved similarly to the high molecular weight commercial PCL in term of complex viscosity ($\eta^*$). However, PU (2) elastic modulus (G') remained below G'' (viscous modulus) (Figure SI2

(C)) until high frequency, showing relatively low elasticity/melt-strength of this material. On the opposite, G' exceeded G" at much lower frequency for PU (1) (Figure SI2 (B)), suggesting that it might represent a potential candidate as SMP.

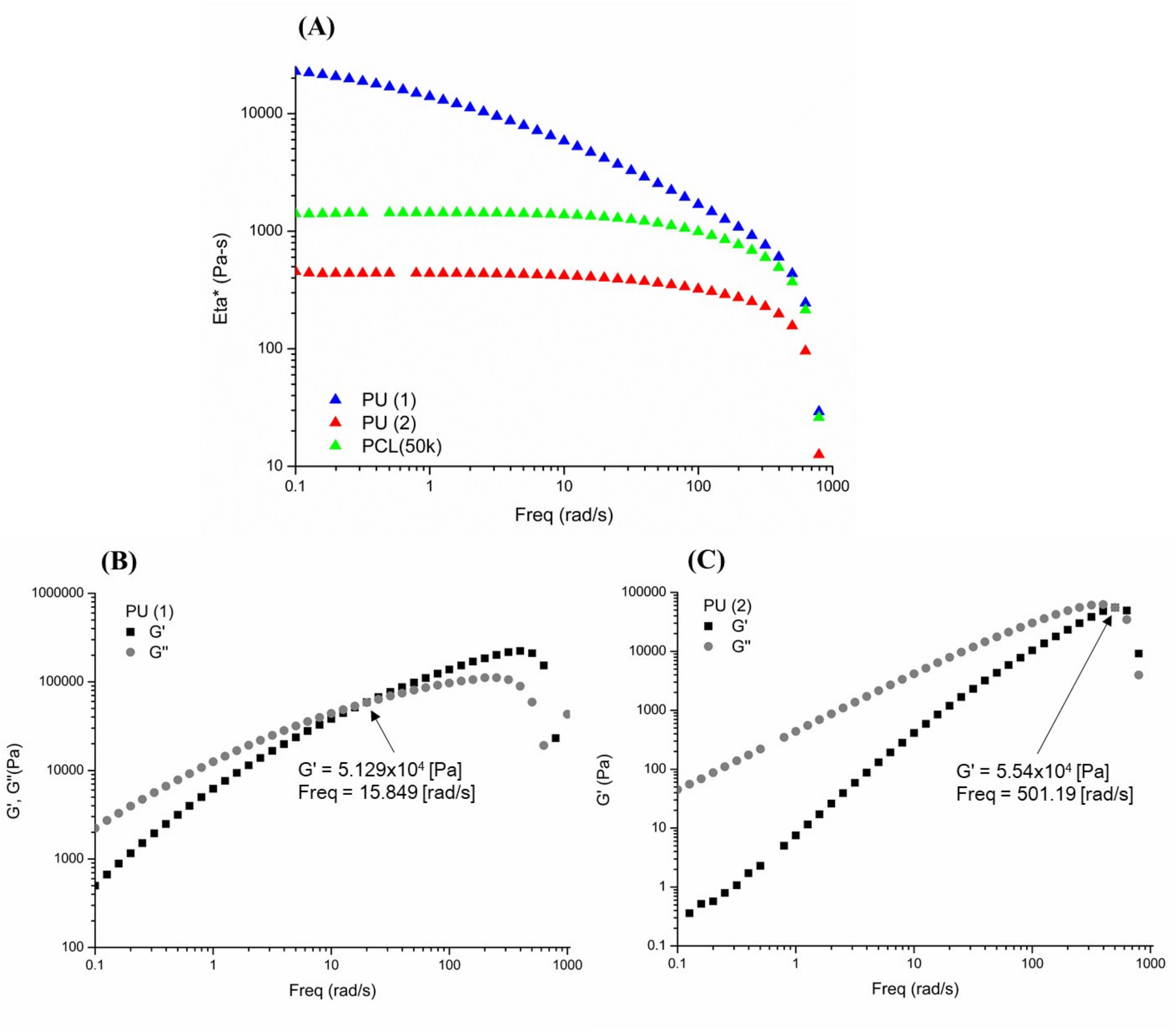


*Figure SI2. Rheological measurements of PU (1), PU (2) and commercially available PCL ($M_n$ of ca. 50 000 g mol$^{-1}$): (A) evolution of the complex viscosity following frequency sweep tests, (B) G' and G'' for PU (1), (C) G' and G'' for PU (2)*
